# Relationship between preoperative hemoglobin levels and length of stay in elderly patients with hip fractures: A retrospective cohort study

**DOI:** 10.1097/MD.0000000000038518

**Published:** 2024-06-21

**Authors:** Ning Zhang, Daxue Zhang, Shuqun Ren, Yan Gao, Weichao Sun, Shiwei Yang

**Affiliations:** a School of Nursing, Anhui Medical University, Hefei, China; b Guangxi University of Chinese Medicine, Nanning, China; c Department of Rehabilitation Medicine, Shenzhen Second People’s Hospital, Shenzhen, China; d Department of Bone and Joint Surgery, Shenzhen Second People’s Hospital, Shenzhen, China; e Teaching Office, Shenzhen Second People’s Hospital, Shenzhen, China.

**Keywords:** elderly, generalized additive model, hemoglobin, hip fracture, length of stay, smooth curve fitting

## Abstract

Globally, hip fractures in elderly individuals are a prevalent and serious issue. Patients typically have a longer length of stay (LOS), which increases the risk of complications and increases hospitalization costs. Hemoglobin (Hb) is a routine blood test that is associated with disease prognosis. This study aimed to investigate the relationship between preoperative Hb and LOS in elderly hip fracture patients and to determine a reliable transfusion threshold. The clinical data of hip fracture patients (aged ≥ 60 years) admitted to the Department of Orthopaedics, Shenzhen Second People’s Hospital, between January 2012 and December 2021 were retrospectively analyzed. Multiple linear regression analysis was used to assess the linear relationship between preoperative Hb and LOS. Smooth curve fitting was performed to investigate potential nonlinear relationships. In the case of discovering nonlinear relationships, a weighted two-piecewise linear regression model was built, and the inflection points were determined using a recursive algorithm. Subgroup analyses were conducted based on age and gender. A total of 1444 patients with an average age of (77.54 ± 8.73) years were enrolled. After adjusting for covariates, a nonlinear relationship was found between preoperative Hb and LOS. The two-piecewise linear regression model revealed an inflection point of 10 g/dL. On the left of the inflection point (Hb < 10 g/dL), the LOS was reduced by 0.735 days for every 1 g/dL increase in Hb (*β* = ‐0.735, 95% confidence interval: ‐1.346 to ‐0.124, *P* = .019). On the right side of the inflection point (Hb > 10 g/dL), the relationship was not statistically significant (*β* = 0.001, 95% confidence interval: ‐0.293 to 0.296, *P* = .992). In elderly hip fracture patients, there is a nonlinear association between preoperative Hb and LOS. However, when Hb levels were <10 g/dL, there was a negative correlation with the LOS. No correlation was observed when Hb levels were >10 g/dL. These findings underscore the importance of timely intervention to manage Hb levels in elderly patients with hip fractures, potentially reducing hospitalization durations and associated complications.

## 1. Introduction

Hip fracture is a prevalent and severe disease among the elderly population.^[1,2]^ It is projected that approximately 4.5 million people worldwide will experience hip fractures by 2050.^[3]^ Hip fractures cause more than 300,000 individuals to be hospitalized in the US each year.^[4]^ Over 500,000 hip fractures occur annually in China, and the incidence is expected to rise at a rate of 25% per decade.^[5]^ Moreover, Hip fractures in the elderly are associated with higher rates of mortality and disability,^[6–9]^ and the associated socio-medical costs are increasing.^[10,11]^ In China, the direct healthcare costs related to hip fractures are expected to reach 15 billion dollars by 2025, nearly double compared to 2018.^[12]^ Currently, the primary therapy for this disease is surgery,^[13]^ but recuperation usually requires a longer length of stay (LOS) for patients. In addition to consuming a significant amount of healthcare resources, a longer LOS increases the risk of postoperative infections, complications, and mortality,^[14]^ thereby increasing the burden on patients and society. Furthermore, LOS is often used as an indicator of care quality and hospital costs.^[15]^ Therefore, improving patient transfer and making the best use of medical resources depend on knowing how to reduce LOS in an efficient, reasonable, and scientific manner.

Hemoglobin (Hb) functions as a diagnostic marker for anemia.^[16,17]^ Its primary function is to transport oxygen to various tissues and organs in the body.^[18]^ Low Hb levels in elderly individuals are frequently associated with factors such as advanced age, trauma, underlying diseases, and malnutrition. According to previous research,^[19–22]^ preoperative anemia affects between 15.3% to 55.4% of patients with hip fractures. Previous research has demonstrated that anemia in elderly hip fracture patients is often linked to negative outcomes, including an increased risk of postoperative infections, increased mortality rates, compromised postoperative functional rehabilitation, and reduced quality of life.^[23–25]^ Recent studies have shown that low Hb is an independent risk factor for prolonged LOS.^[26–28]^ However, this finding remains controversial. Some studies have demonstrated a correlation between low Hb levels in hip fracture patients and prolonged LOS,^[29,30]^ while a retrospective study by Song et al with 1112 participants revealed no association between Hb levels before surgery and hospital stays.^[31]^ To further investigate this relationship, our study retrospectively analyzed the clinical data of elderly patients with hip fractures treated at the Department of Orthopaedics, Shenzhen Second People’s Hospital, and aimed to determine the quantitative correlation between Hb levels at admission and LOS, providing a reference basis for clinical healthcare professionals in formulating treatment strategies.

## 2. Methods

### 2.1. Study design and population

**Figure 1.**
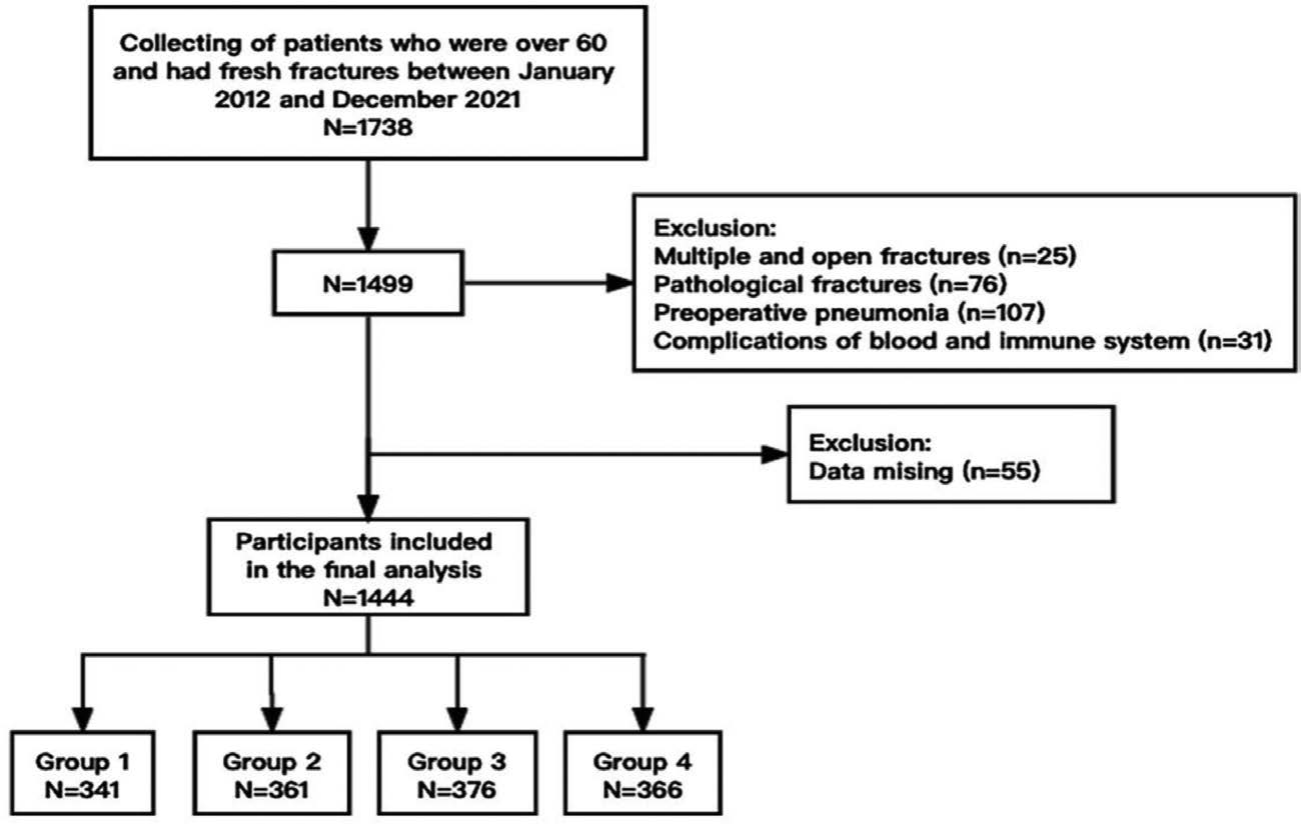
Flowchart of study participants.

Elderly hip fracture patients who underwent surgical treatment at the Department of Orthopedics, Shenzhen Second People’s Hospital, between January 2012 and December 2021 were included in the study. The inclusion criteria were as follows: age ≥ 60 years, X-ray diagnosis of femoral neck fracture or intertrochanteric fracture, surgical treatment, and fresh fracture (within 21 days from the date of injury to hospital admission). The exclusion criteria were multiple or open fractures, pathological fractures, a lung infection diagnosed before surgery, periprosthetic fractures, combined with immune and hematological diseases, and incomplete data. Ultimately, a total of 1444 subjects were included in the study (Fig. 1). Various clinical data, including patient demographics, preoperative laboratory test indices, intraoperative data, and short-term prognosis, were collected for analysis. This study adhered to the principles of the Declaration of Helsinki, obtained ethical approval from the Shenzhen Second People’s Hospital (ethical approval number: 20210620213357012-GZ2022), and was registered with the China Clinical Trial Center (ChiCTR2100047560). Informed consent was waived because the study was retrospective and patient information was anonymized.

### 2.2. Exposure and outcome

The exposure variable in this study was Hb at admission, which was recorded as a continuous variable. Fasting blood samples were collected from hospitalized patients who were required to fast for at least 8 hours. The outcome variable for this study was the LOS (days) for hip fracture patients, defined as the number of days from admission to discharge, which was recorded as a continuous variable.

### 2.3. Covariate selection

Clinical patient data were collected through the hospital’s electronic case system, with double data entry completed using Epidata3.1 software. The demographic data collected for this study included age, gender, body mass index (BMI), smoking status, fracture classification, time from fracture to surgery, and past medical history. Preoperative laboratory data included Hb, lymphocyte count, neutrophil count, white blood cell count (WBC), platelet count, red blood cell distribution width (RDW), serum creatinine, albumin, and blood urea nitrogen. Intraoperative data included information on the anesthesia method, American Society of Anesthesiologists (ASA) classification, operation method, operation duration, and intraoperative blood loss. Short-term prognosis was assessed based on factors such as intensive care unit admission or postoperative pneumonia (POP).

### 2.4. Statistical analysis

In this study, continuous variables with a normal distribution are presented as the mean ± standard deviation. Variables with a skewed distribution are presented as the median (interquartile range). Categorical variables are expressed as numbers and their proportions. To analyze the categorical variables, the chi-square test or Fisher–Freeman–Halton exact test was employed. For normally distributed continuous variables, one-way ANOVA was used, while the Kruskal–Wallis test was applied for skewed continuous variables.

To observe trends in the association between Hb and LOS, the study population was divided into 4 groups (Q1–Q4) based on quartiles of Hb, and the baseline characteristics of each group were compared. Univariate (general linear regression analysis) and multiple linear regression were used to assess the correlations between different Hb levels and LOS, and the results were expressed as *β* and 95% confidence intervals (CI). Three models were created to stepwise adjust for confounding variables that may affect the association between preoperative Hb and LOS. These 3 models are a non-adjusted model (Model I, uncorrected for any covariates), a minimally adjusted model (Model II, corrected only for age and gender), and a fully adjusted model (Model III, further corrected for age, gender, BMI, fracture classification, time from fracture to surgery, comorbidity, hypertension, coronary heart disease, lymphocyte count, WBC count, RDW, albumin, blood urea nitrogen, ASA classification, operation duration, and intraoperative blood loss). Variables with variance inflation factors >10 were excluded from the fully adjusted model.^[32]^ To ensure the robustness of the data analysis, Hb was converted into a categorical variable based on quartiles for sensitivity analysis to verify the results of Hb as a continuous variable. Finally, a generalized additive model and smooth curve fitting (penalty spline method) were used to explore the nonlinear relationship between Hb and LOS, identifying the inflection point of Hb using a recursive algorithm. A log-likelihood ratio test was also performed to compare the one-line linear regression model with the two-piecewise linear regression model. Subgroup analyses were performed using stratified linear regression analyses. Statistical significance was set at *P* < .05.

Analyses were performed using the statistical software packages R (http://www.R-project.org, The R Foundation) and EmpowerStats (http://www.empowerstats.com, X&Y Solutions, Inc., Boston, MA). *P* values <.05 (two-sided) were considered to indicate statistical significance.

## 3. Results

### 3.1. Baseline characteristics of the study participants

A total of 1444 patients who met the study criteria were included. Following the quartile principle, the patients were divided into 4 groups (Q1–Q4) based on their Hb levels. The average age of the patients was 77.54 ± 8.73 years. Overall, 389 (26.9%) were males, and 1055 (73.06%) were females. Among them, 925 (64.1%) had femoral neck fractures, and 519 (35.9%) had intertrochanteric fractures. Compared to the Q1–Q3 group, the Q4 group exhibited a higher proportion of males, younger individuals, a higher proportion of patients with femoral neck fractures, an ASA classification ≤II, and higher lymphocyte counts, neutrophil counts, WBC counts, and albumin levels (*P* < .05). Conversely, the Q4 group had a lower percentage of POP; serum creatinine and blood urea nitrogen levels were lower in the Q4 group than in the Q1–Q3 group (*P *< .05) (Table 1).

**Table 1:** Baseline demographic and clinical data (N = 1444).

Hb(g/dL)	Q1 9.11 (1.08)	Q2 11.09 (0.41)	Q3 12.32 (0.34)	Q4 13.89 (0.84)	*P*-value
N	341	361	376	366	
Age (years)	81.18 (8.04)	78.12 (8.56)	76.92 (8.59)	74.23 (8.32)	<.001
Gender (n, %)					<.001
Male	91 (26.69%)	65 (18.01%)	95 (25.27%)	138 (37.70%)	
Female	250 (73.31%)	296 (81.99%)	281 (74.73%)	228 (62.30%)	
BMI (kg/m^2^)	21.04 (3.14)	21.42 (3.05)	22.27 (3.35)	23.27 (3.01)	<.001
Smoking status (n, %)	17 (4.99%)	12 (3.32%)	18 (4.79%)	20 (5.46%)	.555
Classification of fracture (n, %)					<.001
Femoral neck fracture	124 (36.36%)	214 (59.28%)	285 (75.80%)	302 (82.51%)	
Intertrochanteric fracture	217 (63.64%)	147 (40.72%)	91 (24.20%)	64 (17.49%)	
Time from fracture to surgery (hours)	95.00 (60.00–167.00)	75.00 (49.00–139.00)	71.00 (48.00–121.00)	77.00 (51.00–138.00)	<.001
Comorbidity	262 (76.83%)	254 (70.36%)	266 (70.74%)	260 (71.04%)	.180
Hypertension (n, %)	170 (49.85%)	157 (43.49%)	183 (48.67%)	184 (50.27%)	
Coronary heart disease (n, %)	52 (15.25%)	50 (13.85%)	50 (13.30%)	38 (10.38%)	.269
Hemiplegia (n, %)	12 (3.75%)	9 (2.65%)	9 (2.58%)	19 (5.72%)	.104
Stroke (n, %)	67 (19.65%)	55 (15.24%)	62 (16.49%)	73 (19.95%)	.260
Parkinson disease (n, %)	8 (2.35%)	12 (3.32%)	7 (1.86%)	6 (1.64%)	.434
Diabetes mellitus (n, %)	88 (25.81%)	73 (20.22%)	72 (19.15%)	93 (25.41%)	.060
Lymphocyte count (×10^9^/L)	1.09 (0.79–1.44)	1.18 (0.91–1.56)	1.19 (0.97–1.61)	1.31 (1.01–1.63)	<.001
Neutrophil count (×10^9^/L)	6.37 (4.80–8.46)	6.62 (4.93–8.31)	6.98 (5.32–8.58)	7.29 (5.44–9.59)	<.001
WBC count (×10^9^/L)	8.80 (3.38)	8.87 (2.86)	9.34 (2.88)	9.75 (2.98)	<.001
Platelet count (×10^9^/L)	211.01 (81.77)	208.32 (81.57)	208.53 (75.02)	204.57 (67.15)	.732
RDW (%)	0.14 (0.02)	0.13 (0.01)	0.13 (0.02)	0.13 (0.01)	<.001
Serum creatinine (µmol/L)	73.00 (58.70–98.50)	63.30 (52.00–78.30)	62.15 (51.08–75.00)	63.40 (52.92–77.00)	<.001
Albumin (g/L)	35.20 (5.12)	37.95 (3.58)	38.80 (3.42)	40.06 (4.06)	<.001
Blood urea nitrogen (mmol/L)	6.30 (4.15–8.40)	4.90 (3.17–6.63)	4.80 (2.96–6.50)	4.70 (2.85–6.10)	<.001
Anesthesia method (n, %)					.334
General anesthesia	248 (72.73%)	277 (76.73%)	295 (78.46%)	280 (76.50%)	
Non-general anesthesia	93 (27.27%)	84 (23.27%)	81 (21.54%)	86 (23.50%)	
ASA classification (n, %)					<.001
≤II	103 (30.21%)	165 (45.71%)	178 (47.34%)	178 (48.63%)	
≥III	238 (69.79%)	196 (54.29%)	198 (52.66%)	188 (51.37%)	
Operation method (n, %)					<.001
Internal fixation	195 (57.18%)	161 (44.60%)	129 (34.31%)	114 (31.15%)	
Hip replacement	146 (42.82%)	200 (55.40%)	247 (65.69%)	252 (68.85%)	
Operation duration (min)	82.87 (38.12)	87.01 (38.67)	82.55 (31.88)	87.39 (34.30)	.125
Intraoperative blood loss (mL)	200.00 (100.00–300.00)	200.00 (100.00–300.00)	200.00 (100.00–300.00)	200.00 (100.00–400.00)	<.001
ICU admission (n, %)	12 (3.52%)	6 (1.66%)	8 (2.13%)	5 (1.37%)	.210
POP (n, %)	34 (9.97%)	22 (6.09%)	19 (5.05%)	16 (4.37%)	.011
LOS (days)	11.00 (8.00–16.00)	10.00 (8.00–14.00)	10.00 (8.00–16.00)	10.00 (7.00–15.00)	.303

ASA = American Society of Anesthesiologists, BMI = body mass index, Hb = hemoglobin, ICU = intensive care unit, LOS = length of stay, POP = postoperative pneumonia, RDW = red blood cell distribution width, WBC = white blood cell.

### 3.2. Univariate analysis

General linear regression analysis was used for univariate analysis. The results (Table 2) indicated that 16 factors were significantly associated with prolonged LOS (days) in elderly hip fracture patients. These factors included age, time from fracture to surgery, comorbidities, hypertension, coronary heart disease, hemiplegia, stroke, Parkinson disease, RDW, albumin, ASA classification ≥III, hip replacement, operation duration, intraoperative blood loss, intensive care unit admission, and POP (*P* < .05).

**Table 2:** Univariate analysis of prolonged LOS.

Variables	Statistics	*β* (95% CI)	*P*-value
Age (years)	77.54 ± 8.73	0.06 (0.02, 0.11)	.002
Gender (n, %)			
Male	389 (26.94%)	0	
Female	1055 (73.06%)	‐0.08 (‐0.88, 0.73)	.851
BMI (kg/m^2^)	22.02 ± 3.25	0.09 (‐0.02, 0.20)	.093
Smoking (n, %)	67 (4.64%)	‐0.36 (‐2.06, 1.34)	.678
Classification of fracture (n, %)			
Femoral neck fracture	925 (64.06%)	0	
Intertrochanteric fracture	519 (35.94%)	0.11 (‐0.64, 0.85)	.778
Time from fracture to surgery (hours)	125.38 ± 262.26	0.00 (0.00, 0.00)	.007
Comorbidity	1042 (72.16%)	1.86 (1.07, 2.65)	<.001
Hypertension (n, %)	694 (48.06%)	1.92 (1.21, 2.63)	<.001
Coronary heart disease (n, %)	190 (13.16%)	2.25 (1.20, 3.30)	<.001
Hemiplegia (n, %)	49 (3.65%)	2.27 (0.28, 4.26)	.025
Stroke (n, %)	257 (17.80%)	2.40 (1.47, 3.32)	<.001
Parkinson disease (n, %)	33 (2.29%)	6.08 (3.71, 8.45)	<.001
Diabetes mellitus (n, %)	326 (22.58%)	0.69 (‐0.17, 1.54)	.115
Hb (g/dL)	11.65 ± 1.88	‐0.18 (‐0.37, 0.01)	.059
Lymphocyte count (×10^9^/L)	1.29 ± 0.59	0.38 (‐0.22, 0.99)	.211
Neutrophil count (×10^9^/L)	7.25 ± 3.41	0.08 (‐0.03, 0.18)	.158
WBC count (×10^9^/L)	9.20 ± 3.04	0.09 (‐0.03, 0.21)	.125
Platelet count (×10^9^/L)	208.06 ± 76.44	0.00 (‐0.00, 0.00)	.905
RDW (%)	0.13 ± 0.02	34.61 (12.70, 56.52)	.002
Serum creatinine (µmol/L)	81.17 ± 82.19	0.00 (‐0.00, 0.01)	.375
Albumin (g/L)	38.06 ± 4.44	‐0.11 (‐0.19, ‐0.03)	.009
Blood urea nitrogen (mmol/L)	5.60 ± 3.75	‐0.07 (‐0.17, 0.03)	.149
Anesthesia method (n, %)			
General anesthesia	1100 (76.18%)	0	
Non-general anesthesia	344 (23.82%)	0.17 (‐0.67, 1.00)	.699
ASA classification (n, %)			
≤II	624 (43.21%)	0	
≥III	820 (56.79%)	2.54 (1.83, 3.25)	<.001
Operation method (n, %)			
Internal fixation	599 (41.48%)	0	
Hip replacement	845 (58.52%)	1.53 (0.81, 2.25)	<.001
Operation duration (min)	84.97 ± 35.81	0.03 (0.02, 0.04)	<.001
Intraoperative blood loss (mL)	246.46 ± 195.38	0.00 (0.00, 0.01)	.001
ICU admission (n, %)	31 (2.15%)	11.12 (8.73, 13.51)	<.001
POP (n, %)	91 (6.30%)	6.83 (5.40, 8.25)	<.001

ASA = American Society of Anesthesiologists, BMI = body mass index, CI = confidence intervals, Hb = hemoglobin, ICU = intensive care unit, LOS = length of stay, POP = postoperative pneumonia, RDW = red blood cell distribution width, WBC = white blood cell.

### 3.3. Multiple linear regression analysis of preoperative Hb and LOS

This study investigated the association between Hb and LOS (days) using different covariate adjustment strategies. For sensitivity analysis, preoperative Hb was transformed into a categorical variable based on quadratic grouping, and the *P*-value for the trend test was calculated (Table 3).

**Table 3:** Multiple linear regression analysis of the association between preoperative Hb and LOS in different models.

Exposure	Model I *β* (95%CI) *P*-value	Model II *β* (95%CI) *P*-value	Model III *β* (95%CI) *P*-value
Hb g/dL	‐0.18 (‐0.37, 0.01) .059	‐0.11 (‐0.31, 0.09) .280	‐0.17 (‐0.41, 0.07) .156
Hb quartile			
Q1	Ref	Ref	Ref
Q2	‐1.01 (‐2.03, 0.01) .054	‐0.82 (‐1.85, 0.21) .119	‐0.74 (‐1.78, 0.31) .167
Q3	‐0.42 (‐1.43, 0.59) .414	‐0.16 (‐1.19, 0.86) .754	‐0.11 (‐1.22, 1.01) .848
Q4	‐0.88 (‐1.90, 0.14) .090	‐0.46 (‐1.53, 0.60) .392	‐0.62 (‐1.83, 0.60) .322
*P* for trend	.223	.696	.589

Model I: unadjusted model.

Model II: adjusted for age and gender.

Model III: adjusted for age, gender, BMI, classification of fracture, time from fracture to surgery, comorbidity, hypertension, coronary heart disease, lymphocyte count, WBC, RDW, albumin, blood urea nitrogen, ASA classification, operation duration, intraoperative blood loss.

CI = confidence intervals, Hb = hemoglobin, RDW = red blood cell distribution width, WBC = white blood cell.

### 3.4. Nonlinear association between preoperative Hb and LOS

**Figure 2.**
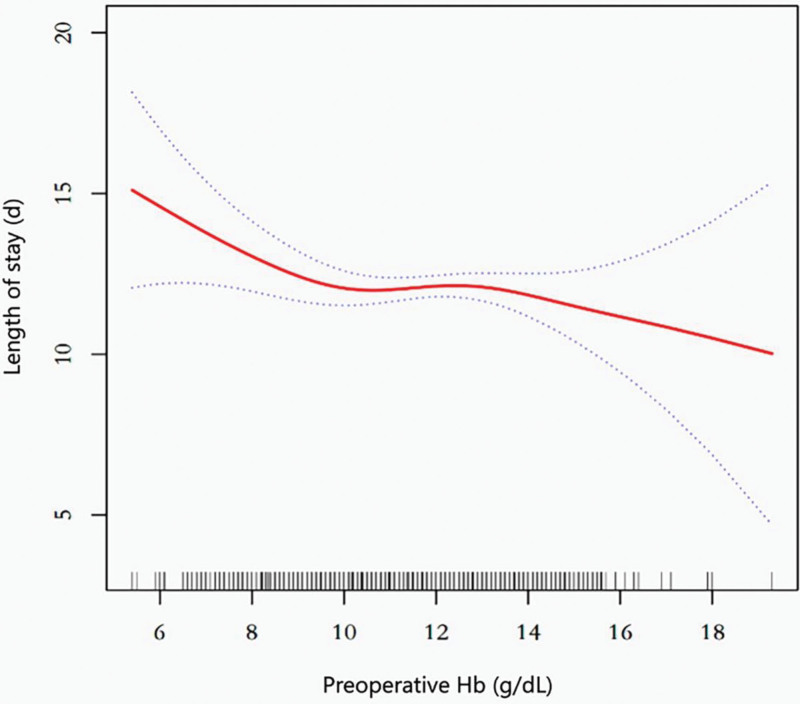
The correlation between preoperative Hb and LOS in patients with hip fracture. Hb = hemoglobin, LOS = length of stay.

The smoothed spline curve in Figure 2 revealed a nonlinear relationship between Hb and LOS. After adjusting for covariates, the two-piecewise linear regression model identified an inflection point at 10 g/dL. On the left side of the inflection point, there was a significant decrease in the LOS of 0.735 days for every 1 g/dL increase in Hb (*β* = ‐0.735, 95% CI: ‐1.346 to ‐0.124, *P* = .019). There was no statistically significant effect on the right side of the inflection point (*β* = 0.001, 95% CI: ‐0.293 to 0.296, *P* = .992) (Table 4).

**Table 4:** Nonlinearity explanation of preoperative Hb and LOS using the two-phase linear model.

	LOS *β* (95% CI) *P*-value
Model 1	
One line effect	‐0.172 (‐0.410, 0.065) .156
Model 2	
Inflection point	10 g/dL
<Inflection point	‐0.735 (‐1.346, ‐0.124) .019
>Inflection point	0.001 (‐0.293, 0.296) .992
*P* for log-likelihood ratio test	.049

Effect: LOS.

Cause: Hb.

Adjusted for age, gender, BMI, classification of fracture, time from fracture to surgery, comorbidity, hypertension, coronary heart disease, lymphocyte count, WBC, RDW, albumin, blood urea nitrogen, ASA classification, operation duration, intraoperative blood loss.

CI = confidence intervals, LOS = length of stay, RDW = red blood cell distribution width, WBC = white blood cell.

### 3.5. Stratified analyses

Stratified linear regression analyses revealed a statistically significant difference among patients who were consistent with the following variables: being female, nonsmoking, having a BMI ranging from 13.3 kg/m^2^ to 20.7 kg/m^2^, experiencing a period from fracture to surgery between 4 to 61 hours, having an intertrochanteric fracture, having a platelet count between (174–219) × 10^9^/L, having a blood urea nitrogen level between (3.82–6.1) mmol/L, and internal fixation (*P* < .05) (Table 5).

**Table 5:** Stratified linear regression analyses of the association between preoperative Hb and LOS.

Variables	(n, %)	*β* (95%CI)	*P*-value
Age (years)			
60–73	472	‐0.19 (‐0.54, 0.15)	.272
74–81	457	‐0.17 (‐0.56, 0.22)	.399
82–108	515	‐0.03 (‐0.33, 0.26)	.822
Gender (n, %)			
Male	389	‐0.03 (‐0.37, 0.31)	.853
Female	1055	‐0.28 (‐0.51, ‐0.04)	.020
BMI (kg/m^2^)			
13.3–20.7	437	‐0.52 (‐0.93, ‐0.11)	.014
20.8–22.9	521	‐0.12 (‐0.41, 0.17)	.407
22.9–35.6	486	‐0.07 (‐0.41, 0.26)	.668
Smoking status (n, %)			
No	1377	‐0.20 (‐0.40, ‐0.01)	.044
Yes	67	0.15 (‐0.68, 0.98)	.723
Classification of fracture (n, %)			
Femoral neck fracture	925	‐0.06 (‐0.32, 0.20)	.661
Intertrochanteric fracture	519	‐0.40 (‐0.74, ‐0.06)	.020
Time from fracture to surgery (hours)			
4–61	473	‐0.27 (‐0.54, ‐0.01)	.045
62–115	485	0.03 (‐0.31, 0.38)	.848
116–8883	486	‐0.13 (‐0.46, 0.19)	.426
Hypertension (n, %)			
No	750	‐0.24 (‐0.49, 0.01)	.062
Yes	694	‐0.14 (‐0.42, 0.14)	.342
Diabetes mellitus (n, %)			
No	1118	‐0.19 (‐0.42, 0.03)	.087
Yes	326	‐0.15 (‐0.50, 0.21)	.416
Platelet count (× 10^9/L)			
49–173	480	0.07 (‐0.23, 0.37)	.631
174–219	482	‐0.59 (‐0.95, ‐0.23)	.002
220–999	482	‐0.10 (‐0.43, 0.23)	.547
Blood urea nitrogen (mmol/L)			
0.98–3.8	475	‐0.03 (‐0.37, 0.31)	.852
3.82–6.1	481	‐0.50 (‐0.88, ‐0.13)	.009
6.2–68	488	‐0.15 (‐0.44, 0.14)	.327
Anesthesia method (n, %)			
General anesthesia	1100	‐0.11 (‐0.32, 0.10)	.294
Non-general anesthesia	344	‐0.38 (‐0.81, 0.04)	.077
Operation method (n, %)			
Internal fixation	599	‐0.40 (‐0.66, ‐0.14)	.003
Hip replacement	845	‐0.18 (‐0.45, 0.09)	.196
Operation duration (min)			
20–69	472	‐0.28 (‐0.57, 0.01)	.060
70–89	445	‐0.26 (‐0.54, 0.02)	.073
90–375	527	‐0.10 (‐0.47, 0.27)	.602

Effect: LOS.

Cause: Hb.

BMI = body mass index, CI = confidence intervals, Hb = haemoglobin, LOS = length of stay.

## 4. Discussion

This study revealed a nonlinear relationship between preoperative Hb and LOS in elderly patients with hip fractures. After fully adjusting for covariates (model III), the LOS decreased by 0.735 days for every 1 g/dL rise in Hb when the preoperative Hb concentration was <10 g/dL (*β* = ‐0.735, 95% CI: ‐1.346 to ‐0.124, *P* = .019). However, when Hb was >10 g/dL, an increase in Hb no longer reduced the risk of prolonged LOS, even though the patients remained mildly anemic (*β* = 0.001, 95% CI: ‐0.293 to 0.296, *P* = .992). These findings suggest that in elderly patients with hip fractures, when the preoperative Hb is <10 g/dL, it is necessary to improve the patient’s physical condition to reduce LOS.

Low Hb levels are a common and significant problem in elderly patients with fractures.^[33]^ Previous studies have shown that approximately one-third of patients have anemia before surgery.^[34,35]^ Zhou ZK et al revealed that the prevalence of preoperative anemia was as high as 29.2% and 45.35% after total hip arthroplasty and femoral head replacement, respectively.^[36]^ Anemia has been identified as a significant predictor of postoperative complications and mortality.^[37,38]^ Our study revealed that preoperative anemia is an independent risk factor for prolonged LOS in elderly hip fracture patients. A study of 589 total elbow arthroplasty patients, using the ACS-NSQIP database, discovered that anemia was a significant predictor of prolonged LOS, with the proportion of patients with prolonged LOS increasing significantly with increasing levels of anemia.^[39]^ In another case-control study involving 1259 patients with hip fractures,^[40]^ Cao H et al reported that Hb was a significant predictor of prolonged LOS (OR = 1.920, 95% CI: 1.045 to 3.529, *P* = .036). However, in an earlier study of 317 elderly hip fracture patients, no correlation between Hb and LOS was found after adjusting for age and gender (*P* = .130).^[41]^ This inconsistency in results may be due to differences in sample size, adjustment variables, and the fact that previous studies did not use nonlinear fitting methods to explore the relationship between Hb and LOS.

The basic biological requirements for fracture repair include adequate Hb, growth factors, and pro-inflammatory factor levels to meet the needs of bone tissue and osteoblasts.^[42]^ Anemic patients have a significantly higher rate of nonunion and failure of fracture fixation.^[25]^ This is because anemia restricts oxygen delivery to tissues and organs, leading to cellular hypoxia and disturbances in intracellular homeostasis. Ultimately, it decreases adenosine triphosphate concentrations and causes cell death.^[43,44]^ Cheng Q et al have demonstrated a positive correlation between endothelial progenitor cells (EPCs) and bone mass.^[45]^ EPCs are capable of secreting bone morphogenetic proteins and other osteoblasts, which promote osteoblast proliferation and differentiation, thus accelerating fracture healing.^[46,47]^ However, anemia reduces the number and function of EPCs, which can impair fracture healing.^[48]^ A study involving 436 patients with distal femur fractures revealed that patients with low Hb had a 4.4-fold increased risk of fracture nonunion compared to those with normal Hb (OR = 5.4, 95% CI: 1.69 to 17.29, *P* = .005).^[49]^ Furthermore, studies have shown that patients with anemia have lower numbers of CD4+ T cells, IgG memory B cells, and plasma cells,^[50]^ which play crucial roles in regulating the immune response. Araújo-Pereira M et al demonstrated that anemia results in increased production of interleukin-6 by the body.^[51]^ Excessive interleukin-6 production exacerbates the inflammatory response, suppresses immune cell function, negatively affects patient prognosis, and prolongs the LOS.

In elderly patients with hip fractures, a low preoperative Hb is associated with prolonged LOS.^[52]^ Low Hb is a common and modifiable risk factor.^[53]^ Increasing Hb levels can promote fracture healing, shorten hospital stays, reduce the risk of postoperative complications and death, improve resource efficiency, and alleviate economic burden.^[54]^ Methods such as treating the primary disease, using erythropoietin, and iron supplementation can effectively increase Hb levels.^[55]^ However, these methods often take time, and patients may face challenges in waiting long enough for surgical treatment. Blood transfusion is a useful approach to rapidly correct anemia by increasing Hb levels. Nevertheless, it is important to note that studies have shown that allogeneic transfusions may increase long-term mortality and cardiovascular events in postoperative hip fracture patients.^[56]^ Therefore, experts suggest carefully weighing the pros and cons before deciding to proceed with a blood transfusion.^[57]^ Based on our findings, when patients have a preoperative Hb level below 10 g/dL, proactive measures should be taken to improve Hb levels. However, when the preoperative Hb level is above 10 g/dL, Hb is not associated with the LOS. Thus, in such cases, avoiding unnecessary blood transfusions may be more beneficial for the patient outcomes. This study provides a reliable threshold reference for clinical staff to develop transfusion strategies.

This cohort study, spanning a decade and featuring a larger sample size than previous studies, adjusted for numerous variables related to LOS and diligently excluded potential confounders. To provide more detailed and reliable results, a stratified analysis was conducted considering factors such as age, gender, and BMI. Significantly, a generalized additive model was used to examine the relationship between preoperative Hb levels and LOS, revealing a nonlinear pattern. The analysis identified an inflection point at 10 g/dL. This is an extremely important discovery, as previous studies have only observed a linear relationship between these 2 variables.

However, it is important to acknowledge certain limitations in our research. Primarily, our study predominantly focused on elderly individuals aged 60 years and above. It remains uncertain whether similar results can be observed among middle-aged and young individuals under 60 years old. Future studies should aim to include a greater representation of middle-aged and young subjects to further validate our findings. Additionally, as our study was a single-center retrospective study, prospective studies with larger sample sizes are warranted to validate the relationship between preoperative Hb and LOS.

## 5. Conclusion

This study suggests that in elderly hip fracture patients, there was no significant correlation observed between preoperative Hb levels and LOS when Hb levels exceeded 10 g/dL. However, a negative correlation with LOS was observed when preoperative Hb levels were <10 g/dL. These findings indicate the importance of enhancing the physical condition of elderly patients to reduce the LOS in patients with very low Hb levels.

## Acknowledgments

We are very grateful to Professor Chen Xinglin for his guidance on the design of this study, and we are also grateful to the Shenzhen Second People’s Hospital for its financial support.

## Author contributions

**Data curation:** Ning Zhang, Daxue Zhang, Shiwei Yang.

**Formal analysis:** Shuqun Ren.

**Funding acquisition:** Shiwei Yang.

**Investigation:** Ning Zhang.

**Methodology:** Ning Zhang.

**Project administration:** Shiwei Yang.

**Supervision:** Yan Gao, Weichao Sun.

**Writing – original draft:** Ning Zhang.

**Writing – review & editing:** Shiwei Yang.
